# GC-MS Profiling, *In Vitro* Antioxidant, Antimicrobial, and *In Silico* NADPH Oxidase Inhibition Studies of Essential Oil of *Juniperus thurifera* Bark

**DOI:** 10.1155/2022/6305672

**Published:** 2022-09-27

**Authors:** Soufyane Lafraxo, Abdelfattah El Moussaoui, Yousef A Bin Jardan, Azeddin El Barnossi, Mohamed Chebaibi, Soukayna Baammi, Aziz Ait Akka, Khalid Chebbac, Mohamed Akhazzane, Tarik Chelouati, Hiba-Allah Nafidi, Khallouki Farid, Mohammed Bourhia, Amina Bari

**Affiliations:** ^1^Laboratory of Biotechnology and Environment, Agri-Food and Health, Faculty of Sciences Dhar El Mahraz, Sidi Mohammed Ben Abdellah University, P.O. Box 1796 (Atlas), 30000 Fez, Morocco; ^2^Department of Pharmaceutics, College of Pharmacy, King Saud University, Riyadh, Saudi Arabia; ^3^Laboratory of Biomedical and Translational Research, Faculty of Medicine and Pharmacy of Fez, University of Sidi Mohamed Ben Abdellah, BP 1893, Km 22, Road of Sidi Harazem, Fez, Morocco; ^4^African Genome Centre (AGC), Mohammed VI Polytechnic University, Benguerir, Morocco; ^5^Laboratory of Biotechnology, Conservation and Valorisation of Natural Resources, Faculty of Sciences Dhar ElMahraz, Sidi Mohammed Ben Abdallah University, P.O. Box 1796 (Atlas), Fez 30000, Morocco; ^6^Engineering Laboratory of Organometallic and Molecular Materials and Environment, Faculty of Sciences Dhar El Mahraz, Sidi Mohamed Ben Abdellah University, P.O. Box 1796 (Atlas), 30000 Fez, Morocco; ^7^Substance Naturelle Pharmacologie Environnement Modelisation Sante et Qualite de Vie, Fez, Morocco; ^8^Department of Food Science, Faculty of Agricultural and Food Sciences, Laval University, 2325 Quebec City, QC G1V 0A6, Canada; ^9^Biology Department, FSTE, University Moulay Ismail, BP. 609, 52000 Errachidia, Morocco; ^10^Higher Institute of Nursing Professions and Technical Health, Laayoune 70000, Morocco

## Abstract

*Juniperus thurifera* is a native species to the mountains of the western Mediterranean region. It is used in traditional medicine as a natural treatment against infections. The present study aimed to carry out the chemical analysis and evaluate the antioxidant, antimicrobial, as well as *in silico* inhibition studies of the essential oils from *Juniperus thurifera* bark (EOEJT). Chemical characterization of EOEJT was done by gas chromatography (GC-MS). We have performed three antioxidant assays (Reducing power (FRAP), 2, 2-diphenylpicrylhydrazyl (DPPH), and total antioxidant capacity (TAC)) of the EOEJT. We next evaluated the antimicrobial activity against *in silico* study, which was carried out to help evaluate the inhibitory effect of EOEJT against NADPH oxidase. Results of the GC/MS analysis revealed seven major compounds in EOEJT wherein muurolol (36%) and elemol (26%) were the major components. Moreover, EOEJT possessed interesting antioxidant potential with an IC_50_ respectively of 21.25 ± 1.02 *μ*g/mL, 481.02 ± 5.25 *μ*g/mL, and 271 *μ*g EAA/mg in DPPH, FRAP, and total antioxidant capacity systems. Molecular docking of EOEJT in NADPH oxidase active site showed inhibitory activity of *α*-cadinol and muurolol with a glide score of −6.041 and −5.956 Kcal/mol, respectively. As regards the antibacterial and antifungal capacities, EOEJT was active against all tested bacteria and all fungi, notably, against *Escherichia coli* K12 with an inhibition diameter of 21 mm and a MIC value of 0.67 mg/mL, as well as against *Proteus mirabilis* ATCC 29906 with an inhibition diameter of 18.33 ± 1.15 mm and a MIC value of 1.34 mg/mL. A more pronounced effect was recorded for the fungal pathogens *Fusarium oxysporum* MTCC 9913 with inhibition of 37.44 ± 0.28% and MIC value of 6.45 mg/mL, as well as against *Candida albicans* ATCC 10231 with an inhibition diameter of 20.33 ± 1.15 mm and a MIC value of 0.67 ± 0.00 mg/mL. Altogether, these results highlight the importance of EOEJT as a source of natural antibacterial and antioxidant drugs to fight clinically important pathogenic strains.

## 1. Introduction

Morocco is the most biodiverse country in the western Mediterranean region [1]. Within it, the eastern half of the forest formations of the Middle Atlas is a veritable mosaic, with a wide range of forest species including cedar, holm oak, Phoenician juniper as well as *Juniperus thurifera* [2]. The Bouiblane site made up of two distinct regions (Bouiblane I and Bouiblane II) is part of the Sites of Biological and Ecological Interest (SBEI) of the Moroccan Eastern Middle Atlas. This locality stands out by its different agro-climatic conditions, its floristic diversity, and its significant but unquantified rate of endemism, as well as its wide range of plant forms [3]. *Juniperus thurifera* is a variably sized tree that belongs to the family Cupressaceae. This species is native to mountainous areas of the Mediterranean basin's western region [4]. In Morocco, *Juniperus thurifera* grows more particularly in the High and Middle-Atlas Mountains [5, 6]. The two arboreal plants that can grow with it are *Quercus ilex* and *Cedrus atlantica*. Such hardy species may tolerate a wide range of weather conditions and may reach up to 19 meters tall and live for up to 800 years. The *thurifer* covers an estimated area of almost 20 000 hectares. It is the most regressed forest species in Morocco, with a 90 percent regression from its potential range [7].

Different phytoconstituents have been isolated from this species, and include mainly polyphenols, essential oils, and sterols [8, 9]. Such metabolites exhibited potential bioactivities such as anti-inflammatory, and antibacterial agents among other wide therapeutic applications [8–10].

Multidrug-resistant organisms have become a major concern and the situation is far from being mastered. Therefore, the research for antibacterial from natural origins has become a vivid area of exploration. Essential oils are combinations of volatile secondary metabolites that occur naturally in plants, which mostly represent the various smells and scents of a variety of plants [10]. A myriad of published reports has provided evidence of their potent bioactivities as antioxidants, antibacterials, antifungals, and insecticides [8–12].

This study aimed at characterizing the bark essential oils of *Juniperus thurifera* by GC/MS. Next, we conducted the antioxidant potential of essential oils *in vitro* and explore *in silico* as NADPH oxidase inhibitors. Finally, the antibacterial and antifungal effects against drug-resistant microbial strains were also described.

## 2. Materials and Methods

### 2.1. Plant Material

**Figure 1:**
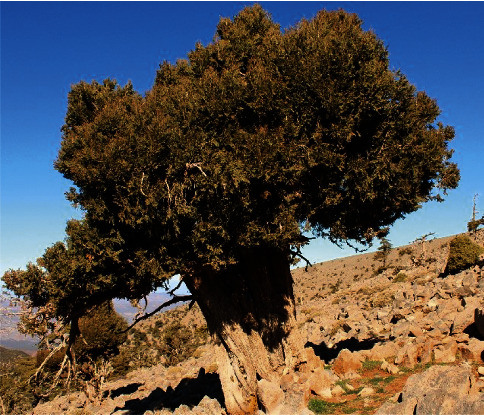
Photograph of *Juniperus thurifera*.

*Juniperus thurifera* (Figure 1) was collected from a mountainous area in the Middle Atlas, Morocco before being identified by a botanist and given the voucher number (FJT/02D20). Next, the bark was isolated from the plant branches, and then cleaned and dried by use of an oven at 35°C before grinding into powder by use of an electric grinder [13].

### 2.2. Chemicals

Ferric chloride (FeCL_3_), sodium phosphate, ammonium molybdate, butylated hydroxytoluene (BHT), 2, 2-diphenylpicrylhydrazyl (DPPH), and 2, 3, 5-triphenyltetrazolium chloride (TTC). Malt extract (ME), sodium chloride (NaCl), trichloroacetic acid (TCA), potassium ferricyanide (K_3_Fe(CN)6), agar, erythromycin, fluconazole, and dimethylsulfoxide (DMSO) were bought from Sigma-Aldrich (St. Louis, MO, USA).

### 2.3. Extraction of EOEJT

Using a Clevenger device, 200 g of the plant powder was immersed in 800 mL of water before being mixed and heated at boiling temperature for two hours. Next, the essential oils (EOEJT) were recovered and placed at 4°C until further use [13, 14].

### 2.4. Chromatographic Analysis of EOEJT by GC/MS

Analysis was done using a gas chromatograph (GCMS-TQ8040 NX Shimadzu brand) with an apolar capillary column (RTxi-5 Sil MS-30.00 m × 0.250 mm ID × 0.250 *μ*m Film thickness). One microliter of EOEJT was used for chromatographic analysis after being diluted in hexane (10 : 100 dilution). The oven temperature program was initially set to 40°C for 2 minutes and followed by 260°C for 10 minutes, then increased by 5°C/min up to 280°C. Finally, the temperature was held at 280°C for 10 minutes. The carrier gas was nitrogen and the flow rate was 1 mL/min. The temperatures of the injector and detector were adjusted to 250 and 280°C. The ionization energy was set to 70 eV, the ion source temperature was set at 200°C, the interface line temperature was 280°C, and the scan mass range was *m*/*z* 40–650.00. Volatile compounds of essential oil were identified by the use of the Kovats index and NIST-MS Search Version 2.0 [13, 15].

### 2.5. Antioxidant Activity of EOEJT

The antioxidant capacity of EOEJTs was assayed by the use of three systems DPPH, TAC test, and FRAP, respectively. EOEJTs were prepared in a dilution series from 1 *μ*g/mL to 1000 *μ*g/mL in methanol; thus, positive controls of quercetin and BHT were used in this evaluation and were prepared under the same conditions as the EOEJTs.

#### 2.5.1. DPPH Test

Free radical powder was prepared in methanol [16,17]. Briefly, 0.004 mg in 100 mL. 100 *μ*L of the sample (EOEJT, BHT, and quercetin) were diluted in methanol before being added to 750 *μ*L DPPH, while a solution consisting of 750 *μ*L DPPH and 100 *μ*L methanol was used as a negative control. The reaction media were incubated for 25 minutes, and the optical density (DO) was measured at *λ* = 517 nm. The antioxidant efficiency was determined according to the following formula:

(1) $$\begin{array}{c} \text{EA}\left(\%\right)=\left(\frac{1-\text{Ds}}{\text{Ct}}\right)\times 100. \end{array}$$

EA: antioxidant efficiency in percent (%); Ds: optical density of sample; Ct: optical density of negative control (White).

#### 2.5.2. FRAP Test

This test was assayed by preparing a reaction medium using phosphate buffer with 200 mM-pH = 6.6 and K_3_Fe(CN)6 (1%) [14,18]. Briefly, 200 µL of each solution was mixed with 50 *μ*L EOEJT before being incubated at 50°C for 30 minutes. To prepare for analysis, the sample was remixed with 200 *μ*L of trichloroacetic acid (10 mg/mL), 500 *μ*L of distilled water (H_2_O), and 100 *μ*L of FeCl_3_ (1 mg/mL). At 700 nm, absorbance was measured against a negative control (50 *μ*L of methanol without EOEJT). The EC-50 effective concentration was used to present the results.

#### 2.5.3. TAC Test by Use of Phosphomolybdenum Method

Briefly, EOEJT was combined with 1 mL of a reagent solution before being heated at 95°C for one hour and then cooled at room temperature for 20 minutes. The optical density was measured at *λ* = 695 nm against a negative control containing 25 *μ*L of methanol without EOEJT, while ascorbic acid was used as a positive reference. Results of the TAC assay were expressed in micrograms equivalent to ascorbic acid per milligram of the sample (*μ*g AAE/mg) [19].

### 2.6. Antimicrobial Capacity

#### 2.6.1. Method for Assessing Antimicrobial Capacity

The disk diffusion assay was used to determine whether or not EOEJT has antimicrobial capabilities [20]. In a nutshell, the double-layer approach was used in order to inoculate bacterial and fungal strains onto Petri plates that contained Mueller–Hinton (MH) agar and malt extract (ME), respectively. The inoculum was prepared from fresh cultures grown in MH and ME media with (0.9% NaCl). Next, Wattman paper discs measuring sex millimeters in diameter were immersed in 20 *μ*L of EOEJT before being placed on Petri dishes surfaces that were previously inoculated with bacteria (10^6^ to 10^8^ CFU/mL) and fungi. Erythromycin and fluconazole served as positive controls, respectively, for bacteria and fungi. Subsequently, Petri dishes inoculated with bacteria and fungi were meticulously incubated at 37°C and 30°C for bacterial and fungi strains, respectively [21]. After 24 hours and 48 hours postinoculation, respectively, inhibition diameters and inhibition percentages were computed for bacterial strains and *C. albicans*. However, after 7 days postinoculation, these calculations were performed for *A. flavus*, *F. oxysporum*, and *A. niger* [13].

#### 2.6.2. Minimum Inhibitory Concentration (MIC)

The MIC of EOEJT against microbial strains was determined using the microdilution method [22]. Briefly, the microplates were prepared in sterile conditions, each sterile 96-well microplate was labeled, and then a 100 *μ*L of EOEJT with a 1 : 10 (*V/V*) dilution of DMSO (10 %) was pipetted into the first column of the plate to perform a series of dilution using sterile MH for bacteria and sterile ME for fungi, and lastly, 30 *μ*L of microbial suspension of each strain (108 CFU/mL) was added to wells. After an incubation period ranging from 48 hours to 7 days for fungi and 24 hours for bacteria at 37°C [13, 22], the MIC was ascertained by using the colorimetric method (TTC 0.2%), [22, 23].

### 2.7. Molecular Docking

#### 2.7.1. Ligand Preparations

For ligand preparations, we used the PubChem database to download all the molecules identified by GC/MS in EOEJT in SDF format. Next, the OPLS3 force field was applied, and the LigPrep tool in Schrödinger's Maestro 11.5 software was used to prepare the structures. Based on the ionization states at pH 7.0 2.0, a total of 32 stereoisomers were generated for each ligand.

#### 2.7.2. Protein Preparation

For receptor preparation, the three-dimensional crystal structure of NADPH oxidase was downloaded from the PDB data bank in PDB format. Protein Preparation Wizard of Schrödinger-Maestro v11.5 was used to add hydrogen atoms to heavy atoms, transform selenomethionine into methionine, and remove water. Next, the OPLS3 force was used for minimization, and fixing the maximum heavy atoms RMSD (root mean square deviation) at 0.30 Å [24].

#### 2.7.3. Receptor Grid Generation

The receptor grid was set at the following coordinates: *X* = 17.584, *Y* = 9.05, and *Z* = 51.541. When the volumetric spacing performed was 20 × 20 × 20, the glide of Schrödinger-Maestro v11.5 was used for SP flexible ligand docking [24].

#### 2.7.4. Receptor Grid Generation

The receptor grid was set at the following coordinates: *X* = 17.584, *Y* = 9.05, and *Z* = 51.541. When the volumetric spacing performed was 20 × 20 × 20, the glide of Schrödinger-Maestro v11.5 was used for SP flexible ligand docking. The ligand was coupled to the grid box produced from protein using “Standard precision” (SP), while SP GScore was used to evaluate the results [24].

#### 2.7.5. Glide Standard Precision (SP) Ligand Docking

In ligand docking, the penalties were applied to noncis/trans amide bonds. The partial charge cutoff was set to 0.15 and the Van der Waals scaling factor and partial charge cutoff were set to 0.80 for each ligand atom, The energy-minimized poses presented by the glide score were used to calculate the final score. The best-docked pose with the lowest glide score value was recorded [25].

## 3. Statistical Analysis

Data were all provided as the mean value and standard deviation of tests performed in triplicate. GraphPad Prism was used to perform statistical analysis. Levine's test was used to verify homogeneity, while Shapiro–Wilks test was used to verify normality. Analysis of variance (ANOVA) flowed by Tukey's HSD test was used to arrange multiple comparisons. *p* values lower than 0.05 were regarded to indicate a statistically significant difference.

## 4. Results

### 4.1. Phytochemical Identification of EOEJTs by GC/MS

**Figure 2:**
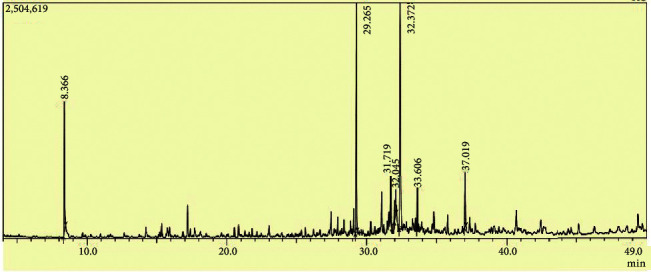
Gas chromatographic profile of EOEJT by GC/MS.

The yield of EOEJTs was about 0.89%, and it is somewhat comparable to what was previously found in the leaves (0.96%) *of Juniperus thurifera* [13]. Seven compounds were identified with a dominance of eudesmane-type cryptomeridiol (37.02%) followed by a cadinane-type muurolol (36.31%) and elemane-type sesquiterpenoid which is elemol (26.93%). The representative GC-MS total ion chromatography (TIC) of EOEJT from the bark of *Juniperus thurifera* is shown in Figure 2 and Table 1.

### 4.2. Antioxidant Activity of EOEJT

**Figure 3:**
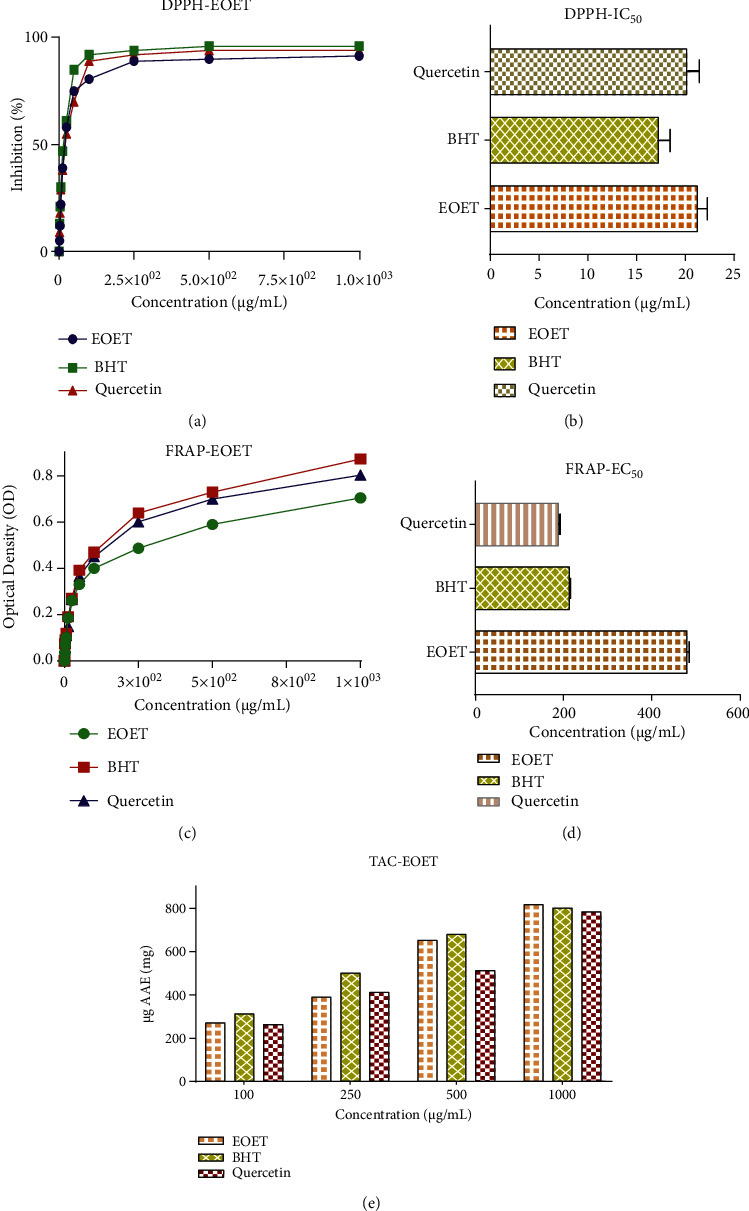
(a, b) Antioxidant capacity using DPPH, (c, d) FRAP method, (e) and total antioxidant capacity.

By use of the DPPH assay, EOEJT showed remarkable antioxidant potency in a dose-dependent manner. The concentration of 50 *μ*g/mL scored an inhibition of about 75%, 85%, and 70%, respectively, for EOEJT, BHT, and quercetin. At a higher concentration, 250 *μ*g/mL, the inhibition percentage was found to be 89.21% (EOEJT), 94% (BHT), and 92% (quercetin), Figure 3(a) Depicts the effectiveness of the tested products, which were determined by the MIC of 50% of free radicals (IC50). The calculated IC_50_ were 21.25 ± 1.02 *μ*g/mL, 17.25 ± 1.20 *μ*g/mL, and 20.15 ± 1.30 *μ*g/mL, respectively, for EOEJT, BHT, and quercetin (Figure 3(b)).

Concerning the FRAP method, EOEJT showed also significant antioxidant effects in a doses-dependant manner compared to the positive controls BHT and quercetin. In this respect, 50 *μ*g/mL of EOEJT or BHT, or quercetin revealed an optical density of 0.33, 0.39, and 0.36, respectively (Figure 3(c)). The EC50 of EOEJT, BHT, and quercetin were 481.02 ± 5.25 *μ*g/mL, 214.08 ± 2.51 *μ*g/mL, and 189.11 ± 2.20 *μ*g/mL, respectively (Figure 3(d)). The antioxidant power of EOEJT was slightly lower than that recorded for *J. thurifera* leaves in previous work with an EC50 of 190 *μ*g/mL [13].

Total antioxidant power was determined using the ammonium phosphomolybdate (TAC) method. Results showed that EOEJT had a good antioxidant capacity since it scored 271 *μ*g AAE/mg, while PHT used as positive control scored 263 *μ*g EAA/mg (Figure 3(e)). These results were comparable to EOs from *Dittrichia viscosa*, which revealed 192 *μ*g AAE/mg as TAC [26]. In addition, the EOs of the leaves of *Lavandula dentata* scored 81.28 *μ*g AAE/mg [27]. EOs from *Withania frutescens* also scored 91 *μ*g AAE/mg [14].

### 4.3. Antibacterial Capacity

**Figure 4:**
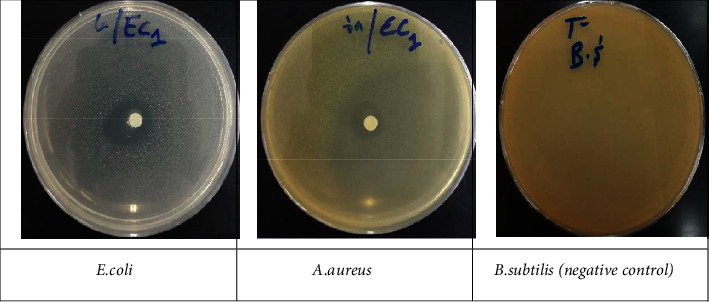
The antibacterial capacity for EOEJT on solid media (disc diffusion method).

**Figure 5:**
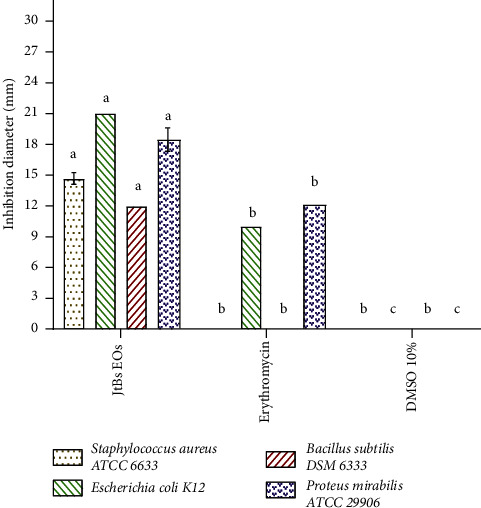
The antibacterial capacity of EOEJT in a solid medium (disc diffusion method), (means ± SD, *n* = 3) marked with the same letter for each strain indicated no significant difference at *p* ≤ 0.05.

EOEJT showed promising antibacterial results when compared to erythromycin, particularly vs. *E. coli* wherein we noted an inhibition zone diameter of 21 ± 0.00 mm and MIC of 0.67 ± 0.00 mg/mL, and against *P. mirabilis* with an inhibition diameter of 18.33 ± 1.15 mm and a MIC of 1.34 ± 0.00 mg/mL (Figure 4, Figure 5 and Table 2). The antibacterial activity of EOEJT may be due to their physicochemical compositions, most importantly the presence of bioactive molecules such as muurolol, elemol, and pinene.

### 4.4. Antifungal Capacity

**Figure 6:**
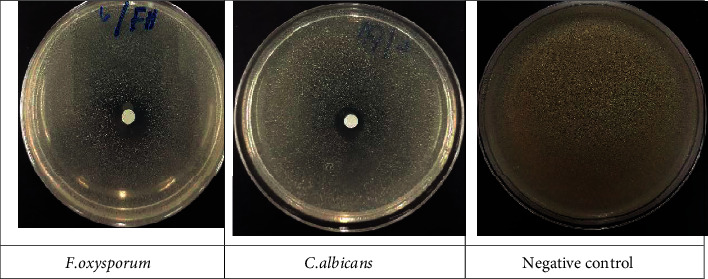
The antifungal capacity for EOEJT on solid media (disc method).

For antifungal susceptibility testing, *in vitro* evaluation of EOEJT against harmful fungal strains was conducted by the use of the diffusion assay. We found a potent antifungal potency with an inhibition percentage of 37.44 ± 0.28% and MIC value of 6.45 ± 0.00 mg/mL against *F. oxysporum* compared to fluconazole (Figure 6 and Table 3). Similarly, EOEJT exhibited significant activity against *C. albicans* with an inhibition zone diameter of 20.33 ± 1.15 mm and a MIC value of 0.67 ± 0.00 mg/mL (Table 3). Besides, EOEJT showed a fungistatic activity vs. *A. niger* and *F. oxusporum* and a fungicidal activity vs. *C. albicans*.

**Figure 7:**
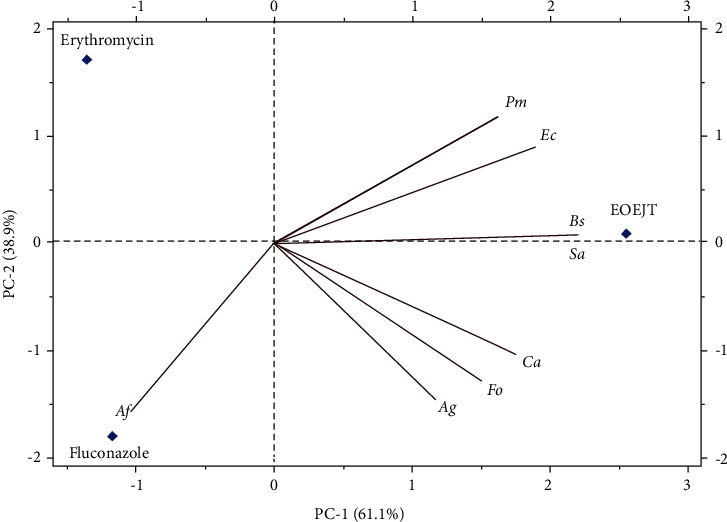
Principal component analysis (PCA) in the C1-C2 plane presents the correlations of antimicrobial activity of EOEJT compared to erythromycin and fluconazole. *Ag*. *A. niger; Af: A. flavus;* Fo*: F. oxysporum; Ca: C. albicans; Sa: S. aureus; Ec: E. coli; Bs: B. subtilis; Pm: P. mirabilis.*

The antimicrobial effect of EOEJT was significant in comparison with one bacterial (erythromycin) and one fungal (fluconazole) specific antimicrobials. The results showed that the EOEJT test was more effective against Gram-negative or Gram-positive bacterial strains. Moreover, the principal component analysis (Figure 7) indicated that all bacterial and fungal strains used for testing showed almost similar sensitivities to EOEJT except for *A. flavus,* which was resistant to both EOEJT and fluconazole.

### 4.5. Molecular Docking

Molecular docking was accomplished to understand the interaction profile between EOEJT and NADPH oxidase. Among all molecules studied, *α*-cadinol, muurolol, gamma-eudesmol, elemol, and *α*-pinene present the highest glide score value of −6.041, −5.956, −5.542, −4.538, and −4.358 kcal/mol, respectively (Table 4).

**Figure 8:**
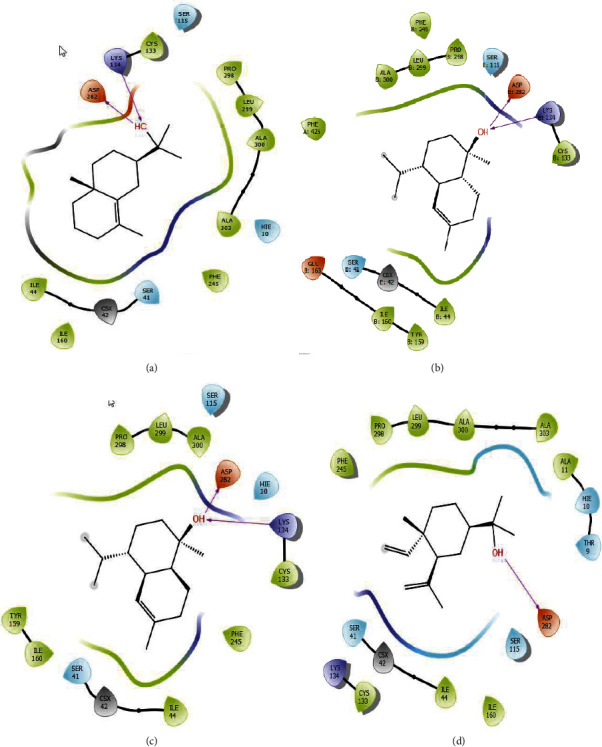
2D diagrams of ligands interactions with the active site of NADPH. (a) *α*-Cadinol; (b) muurolol; (c) *γ*-eudesmol; (d) elemol.

**Figure 9:**
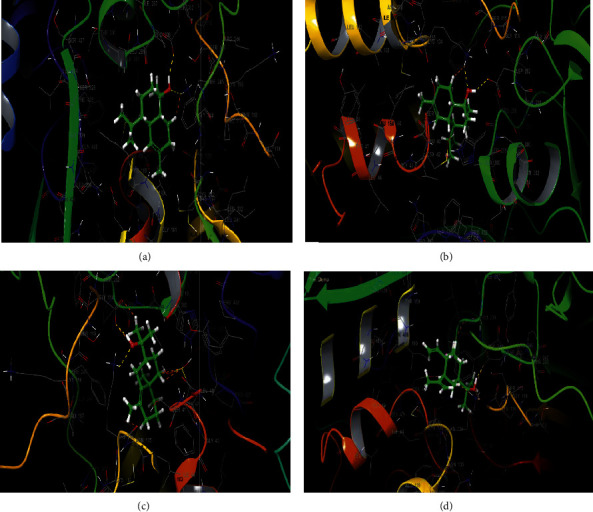
3D diagrams of ligands' interactions with the active site of NADPH. (a) *α*-Cadinol; (b) muurolol; (c) *γ*-eudesmol; (d) Elemol.

Regarding the nature of the bonds between EOEJT and the active site of NADPH oxidase, the molecules which present lower binding energy include *α*-cadinol, *γ*-eudesmol, and muurolol, each one, establishing two hydrogen bonds with the ASP 282 and LYS 134 residues and elemol which formed a single hydrogen bond with residue ASP 282 (Figures 8 and 9).

## 5. Discussion

In the present work, EOs extracted from *Juniperus thurifera* were evaluated for their chemical composition, antioxidant, and antimicrobial effects as no previous work dealing with these pharmacological activities of EOs extracted from *Juniperus thurifera* grows in Morocco to the best of our knowledge. Regarding EOEJT, *Juniperus thurifera* leaves were more qualitatively richer in terpenic compounds and identified 31 compounds with a dominance of *α*-thujene (25%), elemol (12%), and muurolol (12%) [13]. Previously, 24 compounds were reported to account for 99.46% of the mass of *Juniperus thurifera* EOs [28]. Such differences may be due to climatic factors variation (altitude latitude, substrate, etc.), harvest period, organs explored (leaves, stem, and bark), as well as the extraction method used [29]. The assessment of oxidative stress is becoming increasingly significant since this particular type of oxidation has been connected to a wide range of health issues, including rheumatoid arthritis, atherosclerosis, diabetes, cancer, and aging [30–32]. It has been reported that the hydroxyl function present in the phytochemical compositions of EOs is responsible for their antioxidant capacities. Terpenes and phenolic components in EOs are strong antioxidant agents [33–38], which is in agreement with our study. The phytochemical diversity in EOTJ (Table 1), may be responsible for the antioxidant efficiency, whether major or minor compounds may work in synergistic ways [16]. Recent studies have shown the richness of *Juniperus thurifera* of terpinene, which is considered among the compounds that have been shown to increase the antioxidant power [39].

Concerning the antibacterial power, taking a closer look at the published literature supported that muurolol exerted significant antibacterial activity against pathogenic bacteria [40]. Elemol compound, on the other side, has also shown some high biological activity [41]. The active ingredient pinene exhibited significant antibacterial capacity against *P. aeruginosa* and other multidrug-resistant bacteria [42,43]. More particularly, it also had strong antibacterial activity against *S. aureus* and *E. coli* strains [44]. The current results were in agreement with the results reported by Rahhal et al. [45], who showed that *Juniperus thurifera* essential oils exhibited substantial antibacterial capacity against bacteria strains, particularly against *S. aureus* (31.12 ± 3.11 mm of inhibition), *E. coli* (13.23 ± 2.59 mm of inhibition), and *P. aeruginosa* (18.27 ± 2.29 mm of inhibition). Our results are also in agreement with several other studies such as the study by Zeraib et al. [46], which reported that *S. aureus* was highly sensitive to the EO of Algerian *Juniperus thurifera*, and also the study by Bahri et al. [47] demonstrated that the essential oil of *Juniperus thurifera* had potent antibacterial activity against *S. aureus* ATCC 33862 (inhibition zone diameter: 27 mm; minimum inhibitory concentration: 450 *μ*g/mL), *E. coli* ATCC 25922 (inhibition zone diameter: 25.6 mm; minimum inhibitory concentration: 530 *μ*L/mL), and *P. mirabilis* ATCC 7002 (inhibition zone diameter: 18.8 mm).

The interesting antifungal capacity of EOEJT against *A. niger, C. albicans,* and *F. oxysporum* may be due to their active ingredients, especially the high content of bioactive molecules in EOEJT such as muurolol, elemol, and pinene. Many studies have reported that these molecules have a strong antifungal activity, most notably, the study by Chang et al. [48], which showed that muurolol had strong antifungal activity against harmful fungi, whereas elemol was also shown to have strong antifungal activity [41]. For pinene compound, many studies have shown that this compound has significant antifungal activity. Nóbrega et al. [49], showed a significant antifungal action of *α*-pinene against *Candida* spp, and Shi et al. [50], reported also significant antifungal activity of pinene against five plant pathogens including *C. gloeosporioides*, *F. proliferatum*, *A. kikuchiana*, and *Phomopsis* sp.

Many strategies had been devoted to the control of *A. flavus, F. oxysporum, A. niger,* and *C. albicans* using different types of substances, either natural or chemically synthesized. Our results are in agreement with previous reports e.g., the study of Jemli et al. [51], which showed a substantial antifungal activity against *A. alternata*, *F. solani*, *F. oxysporum*, *V. dahlia,* and *R. solani* with a percentage of inhibition ranging from 24% to 92.1%. Lafraxo et al. [13] showed that the essential oil of *Juniperus thurifera* leaves exhibited potent antifungal activity vs. *F. oxysporum* and *C. albicans* at a concentration of 0.095 g/mL.

## 6. Conclusion

This study showed that *Juniperus thurifera* bark essential is rich in terpene compounds, which were extracted and characterized by GC-MS. Additionally, the *Juniperus thurifera* essential oil also had proven antimicrobial, antifungal, and antioxidant capacity. This opens up the possibility of encapsulating this essential oil through complex biotechnology applications with antibiotics to enhance their effects against pathogen resistance. However, before any potential application, further studies dealing with toxicity are warranted.

## Figures and Tables

**Table 1:** Tabulation of the GC-MS analysis of EOEJT.

Peaks	RT	Name	Area (%)	Calculated RI	Literature RI	Molecular structure
1	8.36	*α*-Pinene	14.76	948	939	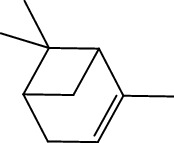
2	29.26	Elemol	26.93	1528	1529	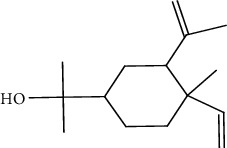
3	31.72	*γ*-Eudesmol	5.23	1664	1662	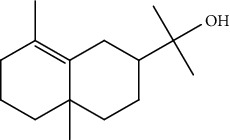
4	32.05	*α*-Cadinol	4.42	1650	1654	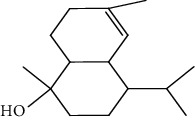
5	32.37	Muurolol	36.31	1640	1642	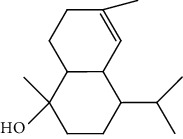
6	33.61	Eicosane	4.55	2007	2009	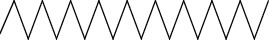
7	37.02	Cryptomeridiol	7.78	1811	1813	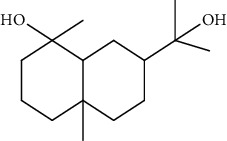
Total 99.98%						

**Table 2:** antibacterial capacity of EOEJT in a liquid medium (microdilution method).

	*Staphylococcus aureus* ATCC 6633 (mg/mL)	*Escherichia coli* K12 (mg/mL)	*Bacillus subtilis* DSM 6333 (mg/mL)	*Proteus mirabilis* ATCC 29906 (mg/mL)
EOEJT	1.34 ± 0,00^a^	0.67 ± 0.00^a^	2.69 ± 0.00^a^	1.34 ± 0.00^a^
Erythromycin	−^b^	3.125 ± 0.00^b^	−^b^	3.125 ± 0.00^b^
DMSO 10%	−^b^	−^c^	−^b^	−^c^

(Mean ± SD, *n* = 3) with different letters in the same column are significantly different (Tow-way ANOVA; Tukey's test, *p* ≤ 0.05).

**Table 3:** antifungal activity of EOEJT.

	Candida albicans ATCC 10231		Aspergillus niger MTCC 282		Aspergillus flavus MTCC 9606		Fusarium oxysporum MTCC 9913	
	Antifungal activity (mm)	MIC (mg/mL)	Antifungal activity (%)	MIC (mg/mL)	Antifungal activity (%)	MIC (mg/mL)	Antifungal activity (%)	MIC (mg/mL)
EOEJT	20.33 ± 1.15^a^	0.67 ± 0.00^a^	21.39 ± 0.57^a^	10.75 ± 0.00^a^	0.00 ± 0.00^a^	−^a^	37.44 ± 0.28^a^	6.45 ± 0.00^a^
Fluconazole	13.00 ± 1.00^b^	7.50 ± 0.00^b^	21.33 ± 1.53^a^	7.5 ± 0.00^b^	0.00 ± 0.00^a^	−^a^	30 ± 0.50^b^	7.5 ± 0.00^a^
DMSO 10%	0.00 ± 0.00^c^	−^c^	0.00 ± 0.00^b^	−^c^	0.00 ± 0.00^a^	−^a^	0.00 ± 0.00^c^	−^b^

(Mean ± SD, *n* = 3) with different letters in the same column are significantly different (Tow-way ANOVA; Tukey's test, *p* ≤ 0.05).

**Table 4:** Docking results of EOEJT in the active site of NADPH.

	Glide Gscore	Glide emodel	Glide energy
*α*-Cadinol	−6.041	−39.399	−28.218
Muurolol	−5.956	−40.689	−28.987
*γ*-Eudesmol	−5.542	−34.926	−26.230
Elemol	−4.538	−34.725	−26.235
*α*-Pinene	−4.358	−20.490	−16.077

## Data Availability

Data used to support the findings are included within the article.
